# Relatives’ experiences of the Boston Psychiatric Rehabilitation approach: A qualitative study

**DOI:** 10.3402/qhw.v9.22918

**Published:** 2014-04-08

**Authors:** Henrika Jormfeldt, Bengt Svensson, Lars Hansson, Petra Svedberg

**Affiliations:** 1 School of Social and Health Sciences, Halmstad University, Halmstad, Sweden; 2 Department of Health Sciences, Lund University, Lund, Sweden

**Keywords:** Mental health services, psychiatric rehabilitation, relatives' experiences, qualitative content analysis

## Abstract

The Boston Psychiatric Rehabilitation (BPR) approach is individualized and characterized by being based entirely on the individual's unique needs and preferences in the areas of working, learning, social contacts, and living environment. Relatives of clients in mental health services influence the client's possibilities for recovery by their everyday relationship. Relatives have, however, traditionally had a subordinated role in the care of their mentally ill family member. The perspective of relatives is an important aspect in the development of new approaches to psychiatric rehabilitation. The purpose of this study was thus to describe and explore relatives’ experiences of the BPR approach. Ten relatives of clients in mental health services taking part in the BPR were interviewed. The interviews were transcribed and analyzed with a qualitative content analysis method to explore relatives’ experiences of the BPR intervention in a county in Sweden. The findings from the interviews could be summarized in the theme “To meet the clients’ needs” consisting of three categories: “Dependence on staffs’ competence,” “Responsibility for user involvement,” and “The necessity for coordination between authorities and caregivers.” The findings suggest that relatives may contribute with important information about clients’ needs related to outcome of care. Relatives’ perspectives may be of importance in future development of BPR. Further research about the relatives’ role in psychiatric rehabilitation is needed as well as studies that compare different kinds of psychiatric rehabilitation from the perspective of relatives.

The Boston Psychiatric Rehabilitation (BPR) approach (sometimes called Choose–Get–Keep Model, CGK) is based on the standards of psychiatric rehabilitation developed by Anthony, Howell, and Danley (1983) at Boston University. The approach has been described as being neither a particular technique nor an intervention but a service within the mental health system (Farkas & Antony, 2010), which aims to promote recovery and the achievement of a meaningful life, rather than simply supporting adaptation to or survival in the community. The BPR is person-centred and characterized by being based completely on the individual's unique needs and preferences (Rogers, Anthony, & Farkas, 2006) in the areas of working, learning, social contacts, and living environment (Anthony, 1992). The BPR is a model for psychiatric rehabilitation that has been developed with the aim of improving the quality of life of persons with severe mental illness through offering extensive person-centred support to strengthen the persons’ ability to take responsibility for his or her life and thereby improve quality of life (Farkas & Antony, 2010). It has been maintained that the perspective of the client and the perspective of the relatives regarding quality of care sometimes represent a conflict of interest (Schröder, Wilde, & Ahlström, 2007). It is thus of importance to gain knowledge of how this extensive person-centred approach is experienced by the service users’ relatives, who may sometimes have different perspectives regarding how the clients’ primary needs are best fulfilled.

The cornerstone of the BPR is, in line with the perspective of service users’, a commitment to a strong partnership between the provider and the client to support the clients’ involvement in planning, carrying trough, and evaluating their achievement of a meaningful life (Farkas & Antony, 2010). The main goal of the BPR is accordingly to support the client in reaching a personal rehabilitation goal in one or more rehabilitation areas. One relevant but not fully understood issue regarding the BPR is the perspective of the relatives. Studies have shown that well-developed cooperation among the client, staff, and his or her relatives support recovery and decrease stress (Ewertzon, Lützén, Svensson, & Andershed, 2010; Gavois, Paulsson, & Fridlund, 2006; Piippo & Aaltonen, 2008). Relatives have mainly described the quality of psychiatric care from their own perspective but have also to a large extent included the patient's perspective as well (Schröder et al., 2007). However, cooperation among the client, staff, and his or her relatives is a challenge for the staff to achieve in mental health services and it is common that relatives are not involved in a person-centred care due to obstacles such as lack of time and lack of competence and professional autonomy among psychiatric nurses (Blomqvist & Ziegert, 2010). Nevertheless, relatives of users in mental health services have been shown to inquire for information about psychiatric illnesses and cooperation in the care regarding their relatives (Schröder et al., 2007).

This study was part of a 2-year follow-up project designed to evaluate the implementation of the BPR in a Swedish county (Svedberg, Svensson, Hansson, & Jormfeldt, 2013). The implementation project was based on the BPR and the purpose of the intervention was to support and guide the client to verbalize and achieve his or her own goals in important life areas such as work or occupation, housing, education, and leisure time. In accordance with the BPR approach, individual and measurable goals and schedules are formulated in the interaction between the client and his or her keyworker to support the former in achieving a satisfying life situation. The intervention comprises three different phases: diagnostic phase, planning phase, and intervention phase, through which the professional and the client work together. All staff at the services where the BPR approach was implemented had completed training in the overall BPR methodology and had also supervised training in providing the different phases of the rehabilitation process.

The relatives play a significant role in the process of recovery among clients in mental health services. The relatives’ unique experiences of psychiatric rehabilitation are thus important when evaluating the outcome of recently developed approaches. No empirical studies have to our knowledge been performed on the subject of relatives’ experiences of this particular approach. The purpose of this study was thus to describe and explore relatives’ experiences of the BPR approach.

## Methods

### Design

The study has an explorative qualitative design using a qualitative content analysis, which is a method that aims to provide knowledge, new insights, and a practical guide to action (Krippendorff, 2004). To produce theoretical representations regarding multi-faceted phenomena for increasing understanding of human experiences, the qualitative content analysis is appropriate (Elo & Kyngas, 2008) even though it has sometimes been considered to be a method that lacks solid philosophical background (Krippendorff, 2004). Qualitative content analyses dealt initially only with the manifest content, but over time the latent content has also been included. It is an appropriate method for identifying variations in terms of similarities and differences in a text (Graneheim & Lundman, 2004). The experiences of being a relative to a person with a severe mental illness are most likely to vary from person to person but also contain something that is common and inclusive. Qualitative content analysis is thus considered a suitable method for this study. An increased understanding of relatives’ views on the effectiveness of the BPR in the clients’ rehabilitation processes could guide policies, interventions, and outcome measures in mental health services.

### Participants

The sample for the present qualitative study consisted of 10 relatives of the group of 49 clients who had completed the 2-year follow-up evaluation project. The criteria for inclusion in this study were that the participant had a family member who was approached in accordance with the BPR approach, during the evaluation project. Six municipal social service units for persons with psychiatric disabilities and one community specialist psychiatric service that operated according to the BPR in a county in Sweden participated in the evaluation. Two of the six municipal social service units only provided vocational rehabilitation. Clients signed an informed consent form, including permission for a relative chosen by the client to participate in an interview, when entering into the evaluation project. The sample of 10 relatives was then selected from the included clients to attain variation in terms of gender and nature of relationship with the clients. All of the requested relatives agreed to participate in the study. The relatives included were six women and four men, representing two mothers, two siblings, one friend, two male cohabitants, one female cohabitant, and two wives. The sample represents all of the services investigated in the county.

### Data collection

Data collection was conducted by telephone interviews during the 2-year follow-up project of the implementation of the BPR between autumn 2009 and December 2010. The interviews started with the question “How would you describe the rehabilitation process and the outcome achieved regarding your next of kin?”. Follow-up questions focused on the relatives’ experiences of the following themes: ability to function in daily life, trust in staff, and competence among staff as well as clients’ self-determination in goal setting and relatives’ degree of involvement and participation. The questions were intended to facilitate an open discussion regarding the relatives’ experiences of the BPR to ensure that no important aspects of these experiences were overlooked. The participants were encouraged to describe their experiences in their own words. The interviews, which lasted between 30 and 60 min, were carried out by two of the authors (H. J., P. S.), who had no involvement in the clients’ care or rehabilitation. The interviewers took careful notes during the interviews and at the end of the interview the detailed notes were read to the interviewee to secure that there had been no misunderstandings regarding the content in the interview.

### Data analysis

The data were analyzed with a qualitative content analysis method (Graneheim & Lundman, 2004). The transcribed interviews were first read through several times to become familiar with the content. Meaning units were then identified, containing aspects related to the purpose of the study. These meaning units were condensed, abstracted, and labelled with a code. The codes were compared to each other to distinguish similarities and differences related to the research question and the content of the text. The findings were finally evaluated by means of discussions between all authors during the analysis process. Examples of how meaning units are condensed, abstracted, and coded; categorized in categories and subcategories; and finally forming the theme “To target the clients’ needs” are given in Table I.

**Table I:** Units of meaning, codes, subcategories, categories and theme regarding relatives’ experiences of the BPR.

Meaning unit	Condensed meaning units	Code	Subcategory	Category	Theme
During the project period, it became a routine for her to do something every day and she was able to do it without being nervous An obvious improvement. (Participant 9)	To get better by practicing a daily routine and reducing nervousness.	Continuity in staff–client relationship and evaluation of goals rehabilitation plan.	Continuity in staff–client cooperation.	Dependence on staffs’ competence.	To meet the clients’ needs.
When my brother is feeling worse the visits by the staff are more frequent and when he feels better the visits become sparser. It is central that staff avoid helping the client to much when the client has the ability to take care of himself. It is important that staff has the ability to adjust the support in relation to individual needs. (Participant 6)	It is important that staff have the ability to vary the support in accordance with the individual needs.	Staffs’ competence regarding supporting the clients’ needs.	Staffs’ ability to individualize client support.		
He could change his schedule himself. Once when he had injured his hand he participated in the rehabilitation program without being able to do so much himself. He was welcome anyway because he needed some place to go during the daytime. … I know that they had meetings where they planned the process step by step towards the rehabilitation goal. (Participant 4)	Being welcome because of the individual need to have some place to go during the daytime.	To be able to participate in goal setting and decision making regarding the schedule.	Clients’ participation and self-determination.	Responsibility for user involvement.	
It would have been better if I'd got more information… I would like to have a clearer role as a relative and be more involved… but I haven't said this so openly. (Participant 3)	It would have been an advantage with more information and to be more involved.	The advantage of relatives getting in contact and having a clear role in the rehabilitation.	Relatives’ participation and involvement.		
She needs more structure, the rehabilitation doesn't demand enough of her. She really needs a therapeutic contact with a psychologist or a cognitive behavioural therapist. She thinks that the process is too slow and she wants to get a job …. (Participant 5)	Staffs’ failure to recognize and fulfil client's need for sufficient help in the rehabilitation process.	Recognizing clients’ needs for interventions from outside the psychiatric rehabilitation program, when needed.	Recognition of clients’ needs.	The necessity for coordination between authorities and caregivers.	
The rehabilitation has been a great help in the contact with for example the social insurance office when my cohabitant got help with explaining that it was too early in her process to start working. (Participant 3)	To get help with explaining that it is too early in the process to start working full time.	The psychiatric rehabilitation program has been helpful in the contact with other caregivers.	Authority to initiate interventions from other caregivers.		

### Ethics

The clients in the 2-year follow-up project gave approval for a relative of their choice to be interviewed about his or her experiences of the BPR when signing the written informed consent to participate in the study. The relatives were informed regarding the study by the interviewer prior to giving their written consent that participation was voluntary and that they could withdraw from the study at any time. The study was performed in accordance with the World Medical Association Declaration of Helsinki. The participants were informed about the purpose and the structure of the study before they gave their written informed consent. The study was approved by the Regional Ethical Review Board, Lund University, Sweden, Dnr 316/2007.

## Results

The findings that emerged from the data could be summarized in the theme “To meet the clients’ needs” consisting of three categories: “Dependence on staffs’ competence,” “Responsibility for user involvement,” and “The necessity for coordination between authorities and caregivers.”

### Dependence on staffs’ competence

The category “Dependence on staffs’ competence” contains the subcategories “Continuity in staff–client cooperation” and “Staffs’ ability to individualize client support.” One expressed experience of the relatives concerned the staffs’ competence and included recognition of continuity in the staff–client relationship. Another expressed aspect of the staffs’ competence was their engagement in the clients’ life. Relatives’ provided examples of when staff had been engaged in the client's needs and the important impact on the client's well-being and growth when staff took responsibility for supporting the client in fulfilling individual needs. Relatives also gave examples of when the lack of staffs’ engagement in the clients’ lives negatively impacted the clients’ development.

#### Continuity in staff – client cooperation

The subcategory Continuity in staff–client cooperation comprises the possibility for practicing tasks every day to gain a routine and learn that one does not need to be nervous with the help of a secure relationship. The subcategory also involves the experiences of the evaluation of goals related to the clients’ rehabilitation plan.

During the project period, it became a routine for her to do something every day and she was able to do it without being nervous …. (Participant 9)

#### Staffs’ ability to individualize client support

The subcategory Staffs’ ability to individualize client support comprises the experience that the staff have the competence to vary the care and support in accordance with the clients’ individual needs. It was considered vital that the support that staff's offer was adjusted to the unique individual and that it was flexible over time and related to the progress during the clients’ process of recovery.

It's vital that staff avoid helping the client too much when he has the ability to take care of himself without any help. It's important that staff are able to vary their support in relation to individual needs. (Participant 6)

### Responsibility for user involvement

The category Responsibility for user involvement contains two subcategories, “Clients’ participation and self-determination” and “Relatives’ participation and involvement.” Relatives spoke of the client being able to participate in goal setting and decision making regarding the schedule and aims in the rehabilitation. The category also includes the advantage of relatives making contact with the services and having a clear role in the rehabilitation when suitable.

#### Clients’ participation and self-determination

The subcategory Clients’ participation and self-determination involves the significance of participation in decision making. The relatives gave examples of their experiences of how the client's lack of participation in the process of rehabilitation could lead to a situation where the client unexpectedly found out that he or she should be moved to a place of work or sheltered workplace while other clients stayed on at the services for a long time without knowing the reason for these differences. The experience of being able to participate in goal setting and decision making regarding the schedule was expressed as:

He could change his schedule himself. Once when he had injured his hand he participated in the rehabilitation program without being able to do so much himself. He was welcome anyway because he needed some place to go during the daytime …. I know that they had meetings where they planned the process step by step towards the rehabilitation goal. (Participant 4)

#### Relatives’ participation and involvement

The subcategory Relatives’ participation and involvement includes the relatives’ experiences of participating in the clients’ process of rehabilitation and contributing to their relatives’ process of recovery. Improved participation would also ease the difficulties that relatives of persons with mental health problems felt when the client did not say much about the rehabilitation process.

It would have been better if I'd got more information … I would like to have a clearer role as a relative and be more involved … but I haven't said this so openly. (Participant 3)

### The necessity for coordination between authorities and caregivers

The category “The necessity for coordination between authorities and caregivers” concerns the relatives’ experiences of recognizing clients’ needs for interventions from outside the psychiatric rehabilitation program. The category comprises the difficulties when the staff does not identify the clients’ needs and the benefit of the psychiatric rehabilitation program in initiating contact with other caregivers when needed. The category embraces the subcategories “Staffs’ competence in assessing the clients’ needs” and “Authority to initiate interventions from other caregivers.”

#### Recognition of clients’ needs

The subcategory Recognition of clients’ needs includes the relatives’ experiences of lack of sufficient support for their ill family member. Relatives also expressed that it is important that the staff are active and take initiative in the relationship with the client in terms of all matters from the first contact through to the planning and follow-up in the rehabilitation process.

She needs more structure, the rehabilitation doesn't demand enough of her. She really needs a therapeutic contact with a psychologist or a cognitive behavioral therapist. She thinks that the process is too slow and she wants to get a job. (Participant 5)

#### Authority to initiate interventions from other caregivers

The subcategory Authority to initiate interventions from other caregivers involves positive experiences of getting help from the psychiatric rehabilitation program concerning the contact with the community support system in terms of housing when the authorities’ decisions do not meet the client's needs. The subcategory also encompasses a desire for improved possibilities for getting help with the coordination of healthcare interventions from other caregivers, and cooperation with the employment agency and the social insurance office. Relatives spoke of situations where the clients’ needs have been neglected because of the staffs’ insufficient competence regarding when to initiate interventions from other caregivers.

The rehabilitation has been a great help in the contact with for example the social insurance office when my cohabitant got help with explaining that it was too early in her process to start working. (Participant 3)

## Discussion

### Discussion of results

The purpose of this study was to describe and explore relatives’ experiences of the BPR approach. The analysis of the relatives’ experiences is summarized in the theme “To meet the clients’ needs,” which consists of three categories: “Dependence on staffs’ competence,” “Responsibility for user involvement,” and “The necessity for coordination between authorities and caregivers.” The categories can be seen as important parts of the theme “To meet the clients’ needs.” The categories are interwoven and no absolute boundaries can be found between them; however, it may be constructive to interpret each dimension of the relatives’ experiences of BPR separately.

The relatives spoke of situations where the staffs’ competence or lack of competence, as well as the clients’ and relatives’ dependence on the staffs’ competence were evident. The relatives in the actual study had the experience of staff having the competence to plan the care in cooperation with the client so that the latter's genuine needs were met in the care. The care was not judged to be constructive by the relatives when it was not experienced as being helpful by the client and complications were perceived when staff were not open to discuss clients’ and relatives’ perspectives in the planning and evaluation of the care. These findings are interesting in the context of the experiences of being a user of psychiatric care, which has been described by former patients as being a structure of control in a “common staff approach” characterized by power and authority (Enarsson, Sandman, & Hellzén, 2011). Researchers have demonstrated the relationship between aspects of the healthcare professionals’ approach and issues of participation as well as clients’ and relatives’ feelings of alienation and powerlessness (Ewertzon et al., 2010; Van der Voort, Goossens, & Van der Bijl, 2007, 2009). Relatives have been shown to need information, accessibility of care, and the ability to talk with a professional as well as needing support in gaining confidence in the ability to cope, support in the appraisal of the behaviour of the client, and in not being judged by professionals (Van der Voort et al., 2007, 2009). The culture and tradition of mental healthcare has predominantly been based on an individualistic view of caring mainly directed by physicians (Blomqvist & Ziegert, 2010). It is thus important that staffs’ attitudes toward relatives are brought to the attention of healthcare staff during their education, since staffs’ attitudes are related to family members’ feelings of alienation (Ewertzon et al., 2010).

The relatives in this study spoke of their experiences with clients’ participation and self-determination in terms of the client being able to participate in goal setting and decision making regarding the care plan. Relatives also expressed that if their participation was improved it would ease difficulties in relation to the client in the rehabilitation process. Participation is practiced in psychiatric rehabilitation by the use of shared decision making, which is a relatively new and somewhat controversial concept in the mental health field (Forrest, 2004). Studies have shown that the clients’ process of recovery and the relatives’ experiences in the process have a mutual influence on each other and form part of a cumulative process (Tranvåg & Kristoffersen, 2008). Negative effects, among relatives, of feelings of being overlooked or turned away by healthcare staff may increase the relatives’ burden and reduce their ability to master the strain and lead to negative pre-understandings of the future, which can possibly counteract a positive change in the client and his or her family (Tranvåg & Kristoffersen, 2008). Although not evident in this study, the relatives’ presence and participation have also been seen to generate unwanted effects (Piippo & Aaltonen, 2008). One risk is when the staffs are not able to listen to each side in a balanced way, resulting in the patients feeling excluded. Another risk is that the relatives might influence the staffs’ views about the clients’ situation if the staffs pay attention to the relatives more than to the patient (Piippo & Aaltonen, 2008). Staffs’ attitudes of perceiving relatives’ participation as a help or a hindrance have been highlighted as important issues regarding the role staff play in encouraging participation among both clients and relatives in mental health services (Goodwin & Happell, 2007).

The necessity of coordination between authorities and caregivers embraces the relatives’ experiences of difficulties in terms of when the staffs do not identify the clients’ needs. Relatives speak of their experiences of staff actively initiating a relationship with the client and the need for improved possibilities for getting help with coordination of healthcare interventions and cooperation with the employment agency and the social insurance office. The findings from an interview study with the clients involved in the BPR intervention suggest that clients do not always recognize nor are able to verbalize their goals before they have been given the possibility to reflect on their possibilities in collaboration with a trusted person (Jormfeldt, Svensson, Hansson, & Svedberg, 2014). The study regarding the clients’ experiences of the BPR did not reveal the importance of staff having the competence of acknowledging the clients need of support from other caregivers. Relatives may have an important role in recognizing when clients’ needs are not sufficiently recognized. This finding is supported by Piippo and Aaltonen (2008) when stating that through shared discussions among the client, relatives, and caregivers, misunderstandings and incorrect interpretations of the clients’ situation can be avoided.

### Methodological considerations

To assess the trustworthiness of the study findings; credibility, transferability, and dependability, need to be considered (Graneheim & Lundman, 2004; Krippendorff, 2004). Credibility, that is, the confidence in the “truth” of the findings, was addressed by including a strategic sample. When using qualitative content analysis, it is preferable that the sample has a variation regarding sex, age, and experiences of the studied topic to increase the possibility of the research question being answered from different perspectives (Graneheim & Lundman, 2004). The fact that interviewees were collected from a 2-year follow-up evaluation could lead to a less heterogeneous sample. To secure credibility in this study, the interviewed relatives were purposively selected among the sample of the evaluation study to attain a variation of experiences related to BPR with regard to gender and nature of relationship to the receiver of rehabilitation. For the reader to evaluate transferability, that is, the possibility of transferring the results to other contexts, a clear description of the interviewees and their context, data collection, and process of analysis has been provided. Transferability is strengthened through the detailed description of the analysis process, which exemplifies how the original meaning units have been condensed, abstracted, coded, and categorized. The fact that the categories were exemplified with quotations further strengthens transferability. Dependability, that is, the extent to which the same findings will appear under similar circumstances and how well the results can be confirmed by others, was addressed by following the same procedure in every interview and by including dialogue among co-researchers with different professional backgrounds. To secure dependability of the data collected in qualitative research, it is important that the interviewer and interviewees have a mutual understanding about the topic of the interview. The fact that the interviews were completed by telephone and that interviews were not tape recorded may reduce dependability of the results. However, dependability in the present study was strengthened by thorough notes being made during the interviews by the interviewers’ and by the interviewers’ extensive knowledge and capabilities of the topic of the interview and of interviewing. The two authors involved in the interviews had previously worked for 15 years in mental healthcare as mental health nurses and both had extensive experiences of interview practice. Dependability was further strengthened by the authors’ different preconceptions being highlighted and discussed during the process of analysis. A detailed description of analysis increases the possibility for valuing the credibility of the results when using qualitative content analysis (Graneheim & Lundman, 2004).

## Conclusion

The purpose of this study was to describe and explore relatives’ experiences of the BPR approach. The analysis of the relatives’ experiences resulted in three categories: dependence on staffs’ competence, responsibility for participation and self-determination, and the necessity for coordination between authorities and caregivers which can be seen as important parts of the theme “To meet the clients’ needs.” All of the experiences described by the clients’ relatives are relevant and related to mental healthcare and psychiatric rehabilitation in general and the findings thus could serve as a foundation for further research in related research areas. Studies regarding clients’ experiences of psychiatric rehabilitation may not reveal the importance of acknowledging the clients need of support from other caregivers because of the clients’ health status and dependence of the staff. This study supports previous research that suggests that cooperation among the client, staff and his or her relatives supports recovery and decreases stress and the relatives may have an important role in recognizing when clients’ needs are not sufficiently recognized in the care they are offered.

## Implication

The BPR approach involves both a person-centred approach and shared decision-making processes. It is thus important to study the relatives’ perspective so that staff are able to recognize the interest of both the client and his or her relatives as groundwork for a holistic perspective of the clients’ situation. As the holistic understanding of the client is central in mental healthcare in general and most kinds of psychiatric rehabilitation, further research is desirable regarding involvements of relatives in the care planning to target needs of social involvement among persons with severe mental illness. Relatives’ negative feelings of being overlooked or turned away by healthcare staff may increase their burden and reduce their ability to master the strain and stimulate positive changes in the client and in the family. Education of healthcare professionals should thus also target the importance of the relationship between the healthcare professionals and clients’ relatives.
